# Cluster headache: a *quasi*-rare disorder needing a reappraisal

**DOI:** 10.1186/s10194-015-0545-1

**Published:** 2015-06-26

**Authors:** Paolo Martelletti, Dimos-Dimitrios Mitsikostas

**Affiliations:** Department of Clinical and Molecular Medicine, Sapienza University, Rome, Italy; Department of Neurology, Athens Naval Hospital, Athens, Greece

**Keywords:** Cluster headache, Trigeminal autonomic cephalagias, Epidemiology, Burden, Pathophysiology, Treatment

This Editorial introduces the 2015 Open Thematic Series dedicated to Cluster Headache (CH) and other Rare Headaches. For too long researchers have focused their attention on other most popular, sometimes clumsy or foggy forms of headache, overlooking this important and *quasi*-rare headache disorder [1].

Apparently CH represents the most peculiar form among the Trigeminal Autonomic Cephalalgias (TACs). The unbearable periorbital side-locked pain is coupled by ipsilateral cranial autonomic symptoms (conjunctival injection, lacrimation and rhinorrhea) and causes a vast disability although the overall burden of this disease has not yet found a complete systematization.

Especially its chronic form, characterized by more days with attacks than not in the year span, represents a strict minority (10 % of CH, 0,01 % overall) and hits 70.000 on a sample of 100 millions of people. Even if these numbers are relatively small the impact of this disease is destructive, thus CH represents a niche worth to be re-focused.

Recently Chronic CH (CCH) refractory to whichever medical therapy (rCCH) has been systematized from a clinical point of view [2].

The personal burden caused by CH, meant as loss of employment, homebound days and disability is higher in women than in men [3].

CH lifetime prevalence is 1/1000 and over the past decades a progressive reduction of the male/female ratio has been reported, being now 2.1:1 [4]. Even if CH is easy to diagnose on the basis of its peculiar features, the timing over the day of the attacks (up to 8), a temporally defined active cluster period (60–90 days) and its seasonal recurrence, only 1/3 of these patients is rightly diagnosed, with an unacceptable delay of 5.3 years and consequently more than 2/3 of them never receive a correct treatment [1, 5, 6].

Actually the current pathophysiological theory of CH is oriented towards a posterior hypothalamic dysfunction [7] but further pharmacological studies shall selectively focus this area. Recently the allele G of the G1246A HCRTR2 polymorphism was been found to be associated with CH, indicating that hypocretin/orexin system’s peptides may be involved in the transmission of pain, in autonomic and neuroendocrine functions, and in the pathogenesis of CH [8, 9].

For too many years sumatriptan, verapamil and corticosteroids have been the cornerstones of CH treatment [1]; we hope that the starting era of monoclonal antibodies against Calcitonin Gene-Related Peptide might include CH as therapeutic target. An ongoing phase 3 RCT on the use of LY2951742 in episodic CH through its preventative subcutaneous administration every 30 days could change the future management of CH [10].

Other approaches to chronic cluster headache are the new mini-invasive and non-invasive neuromodulation techniques. The European Headache Federation recommends caution in using these techniques [11] because only few controlled studies have been carried out yet.

Among these new approaches, Vagal Nerve Stimuation (VNS) and sphenopalatine ganglion stimulation (SPG) seem promising but still classified at Class IV evidence [12, 13]; SPG shows contrasting evidence [14]. All these approaches need to be further confirmed and validated by *ad hoc* RCTs.

Lastly, patients’ education is another important topic to be re-considered: sleep pattern changes, alcoholic beverages, NO-derived cardiovascular drugs and phosphodiesterase inhibitors widely self-prescribed for erectile dysfunction trigger additional bouts during CH active phases [1]. Furthermore, a more diffuse physicians education on CH management should lead to an earlier diagnosis and a more adequate treatment of this headache disorder [15].

Thus we trust that this Open Thematic Series dedicated to Cluster Headache could thicken the attention on a famed and *quasi*-rare headache disorder.
